# Comment on “Micronucleus Evaluation in Exfoliated Human Buccal Epithelium Cells among E-Waste Workers in Payatas, the Philippines”

**DOI:** 10.5696/2156-9614-11.29.210309

**Published:** 2021-02-25

**Authors:** Daniel Araki Ribeiro

**Affiliations:** Department of Biosciences, Federal University of Sao Paulo—UNIFESP, Rua Silva Jardim, 136, Room 332, Vila Mathias, Santos–SP, 11050-020, Brazil, Tel. +55 1332290156 daribeiro@unifesp.br

I read the manuscript recently published by Berame *et al*.^1^ in the *Journal of Health & Pollution* titled “Micronucleus Evaluation in Exfoliated Human Buccal Epithelium Cells among E-Waste Workers in Payatas, the Philippines” with much interest. In this article, the authors detected high frequencies of micronucleus and karyolysis in buccal mucosa cells from individuals exposed to e-waste. However, it is important to properly discuss the scientific approach for a correct understanding of the paper.

According to the Micronucleus Assay Expert Group, there are some confounding factors when performing the micronucleus assay using buccal cells, such as age, smoking habits, and gender.^2–4^ Unfortunately, this was not properly discussed by the authors in the manuscript. For example, it was established that a total of 77.5% of participants (n=31) were smokers in the exposed group, with statistically significant differences (p=0.00) compared to the control group. What were the criteria for categorizing these participants as smokers? This information is very important since it is well established that cigarette smoke is able to induce metanuclear changes in oral mucosa cells,^5–7^ dependent on the type of cigarette as well as nicotine content.^8^

In addition, how many cells were evaluated per individual? This information was not mentioned in the Methods section. It is strongly recommended by the Micronucleus Assay Expert Group to evaluate a minimum of 2,000 cells per volunteer.^3^ The Methods stated that “Slides were stained in 2% Giemsa solution for 5 minutes and washed with distilled water, air dried and then viewed in a binocular microscope.” After that, identification of micronucleus was described, as follows: “Micronucleated cells (*Figure 2a*) are characterized by the presence of the main nucleus and smaller structure denominated micronuclei. Micronuclei have a circle or oval shape with a length between 1/3 and 1/16 of the main nucleus; the intensity of the stain, texture, and plane is equal in both structures.” It is important to highlight that Giemsa stain cannot be used to stain exfoliated cells when using the micronucleus assay since it is not specific for nucleic acids.^3^ In light of the absence of DNA specificity of the stain, micronucleus identification is sometimes impossible due to the presence of some components in the cytoplasm of oral keratinocytes that remember micronuclei, for example, keratohyalin granules, inflammatory cells, or bacteria. This was clearly evidenced in Figures 2a and 2b presented in the manuscript. This suggests rethinking the micronucleus data presented in the manuscript.

As previously described in the manuscript, Tolbert *et al*.^9^ have described several metanuclear changes indicative of cellular death (cytotoxicity) for the buccal micronucleus assay, such as pyknosis, karyorrhexis and karyolysis. The approach is very interesting because cytotoxicity is a potential for bias in the micronucleus assay. For example, if cytotoxicity is increased, the micronucleus frequency decreases because micronucleated cells are lost as a result of cellular death. This should be discussed in the manuscript as well.

I believe that these comments could be useful for better understanding of this important article on the biomonitoring of occupational e-waste exposure.
